# Low level of serum total cholesterol predicts mortality in early-stage multiple system atrophy: a prospective-cohort study

**DOI:** 10.3389/fnut.2025.1663881

**Published:** 2025-10-28

**Authors:** Jingxuan Huang, Lingyu Zhang, Qirui Jiang, Shichan Wang, Yi Xiao, Ningning Che, Junyu Lin, Bi Zhao, Yangfan Cheng, Chunyu Li, Huifang Shang

**Affiliations:** Laboratory of Neurodegenerative Disorders, Department of Neurology, Rare Diseases Center, West China Hospital, Sichuan University, Chengdu, China

**Keywords:** serum total cholesterol, MSA, survival, prospective observational cohort, predictor

## Abstract

**Background:**

Serum total cholesterol (TC) is associated with the risk of multiple system atrophy (MSA). However, the potential impact of the serum TC levels on the mortality of patients with MSA remains to be elucidated. The study aims to clarify the association between baseline level of serum TC and survival of patients with early MSA.

**Methods:**

A total of 364 patients with MSA were recruited and assessed at baseline and follow-up. Patients with MSA were stratified into three groups based on the serum TC tertiles. The role of serum TC on survival was analyzed using Kaplan–Meier survival analysis and Cox regression models. Restricted Cubic Spline regression was employed to investigate the non-linear relationship between serum TC levels and survival.

**Results:**

During a median follow-up period of 4.75 years, the survival duration of patients with MSA was shorter in the lowest serum TC group compared to the other two groups (Log-rank *p* = 0.004). In the multivariable Cox regression model, individuals in the intermediate serum TC group demonstrated a reduced mortality compared to those in the lowest group (HR: 0.47; 95% CIs: 0.23–0.96). There was a non-linear relationship between serum TC level and survival with the lowest risk of death at the value of 4.38 mmol/L.

**Conclusion:**

Serum TC level at baseline negatively correlated with survival of patients with MSA. Serum TC emerges as a significant predictor of mortality in patients with early-stage MSA.

## Background

Multiple system atrophy (MSA) is an adult-onset *α*-synucleinopathy characterized by a poor prognosis (1, 2). The disease progresses rapidly, with patients requiring walking aids within 3 years, transitioning to wheelchair dependence after 5 years, and becoming bedridden within 6 ~ 8 years (3, 4). The median survival time of patients with MSA varies from 6 to 10 years, depending on region and ethnicity (5–7). The pathophysiology of MSA is obscure even though glial cytoplasmic inclusions (GCIs) were regarded as the histopathological hallmark of MSA (8). Therefore, it is necessary to identify early-stage prognostic factors for MSA to elucidate the pathophysiological mechanisms and to explore potential pharmacological interventions in the future.

Cholesterol, an indispensable component of cellular membranes in mammalian cells, has been implicated in various neurodegenerative diseases, including Alzheimer’s disease (AD) (9, 10), Parkinson’s disease (PD) (11, 12), etc. Given that the pathology of MSA primarily affects oligodendrocytes, which are rich in membranes that form the myelin sheath, it is hypothesized that cholesterol may play a significant role in MSA. Furthermore, patients with MSA have been observed to exhibit abnormal serum total cholesterol (TC) levels (13). Some cross-sectional studies suggest that serum TC may serve as a risk factor for MSA (13, 14). Besides, serum TC levels have been associated with critical symptoms such as orthostatic hypotension (OH) in MSA (15).

To date, several cohorts have investigated various predictors of survival in MSA, including neurofilament light chain levels (16), neutrophil-to-lymphocyte ratio (17), and nutritional status (18), etc. However, longitudinal studies examining the predictive value of serum TC on prognosis of MSA are lacking. Therefore, the present study aims to explore the relationship between levels of serum TC at baseline and survival in early stage of patients with MSA.

## Methods

### Study participants and design

This is a prospective, single-center observational study conducted in West China Hospital of Sichuan University from January 2012 to June 2022. Patients with MSA were continuously screened, and early stage of MSA with disease duration less than 3 years was identified. Baseline exclusion criteria included patients with other neurological disorders (such as severe stroke, traumatic brain injury, epilepsy, leukoencephalopathy, brain tumors, etc.), severe liver and kidney function impairment. Besides, all patients had no family history and spinocerebellar ataxia (SCA) genes, including SCA-1, 2, 3, 6, and 7. With regular follow-up, a total of 508 patients with complete hematological information included at baseline. During follow-up appointment, 70 patients with possible MSA, 3 patients with PD, and 2 with progressive supranuclear palsy were excluded. Besides, 69 patients were lost to follow-up. At last, the data at baseline and follow-up of 364 patients with probable MSA were included in the final analysis (19). All patients signed informed consent, and the study was approved by the Ethics Committee of West China Hospital of Sichuan University.

### Baseline indicators measurements assessment

At baseline and follow-up, experienced neurologists collected basic data, including age, sex, body mass index (BMI), smoking and drinking history, etc. and clinical information, including disease duration, age of onset, etc. BMI was calculated by weight (kg)/height^2^ (m). The severity and progression of MSA were evaluated by the Unified Multiple System Atrophy Rating Scale (UMSARS) score with part I to part IV, representing 4 aspects (activities of daily living, motor examination, autonomic examination, and global disability, respectively). The total UMSARS score was calculated by the sum of UMSARS-I and -II scores (20). Orthostatic hypertension (OH) was defined as a reduction of systolic blood pressure (SBP) ≥ 20 mmHg and/or of diastolic blood pressure (DBP) ≥ 10 mmHg within 10 min orthostatic test (21). Symptom onset was recorded as the date of initial presentation of any symptom, including motor and autonomic features (except for erectile dysfunction). Disease duration was considered as the course of the time of symptom onset to assessment. Survival time was defined as the interval between the symptom onset and the date of death, otherwise the interval between the symptom onset and the date of the last follow-up appointment of the surviving patients. MSA-P refers to patients with predominant parkinsonism, and MSA-C refers to predominant cerebellar ataxia (18). Among the comorbidities, diabetes was defined as patients meeting one of the following criteria: (1) having been diagnosed with diabetes or taking hypoglycemic drugs, (2) glycated hemoglobin (HbA1c ≥ 6.5%), and (3) fasting blood glucose (FBG) ≥ 7.0 mmol/L. Patients with hypertension is considered as one of the followings: (1) having been diagnosed with hypertension or taking antihypertensive drugs, (2) SBP ≥ 140 mmHg or DBP ≥ 90 mmHg. Baseline serum TC referred to the levels of serum TC at the time of the patient’s first visit. Fasting venous blood was collected from patients in the early morning following admission to analyze the levels of serum TC and other serological parameters by standard laboratory methods.

### Statistical analysis

Descriptive data were presented in the form of mean ± standard deviation or frequencies (percentage). In the comparison between surviving and deceased patients with MSA, Student’s *t*-test or Wilcoxon rank sum test were used for continuous variables, and Chi-square test or Fisher exact test for categorical variables. According to the distribution of serum TC levels at baseline in patients with MSA, levels of serum TC were further classified into three groups, and the lowest tertile group was defined as the reference group. An analysis of variance (ANOVA) test or Kruskal-Wallis’s test was used to compare continuous variables between different groups regarding the level of serum TC, as appropriate. The cumulative event rates and survival were analyzed by Kaplan–Meier curve, and the Kaplan–Meier hazard ratios (HRs) were evaluated by log-rank test. Cox proportional hazard regression model was used to analyze the relationship between levels of serum TC and survival. In the multivariate Cox proportional hazards regression model, covariates were selected for regression model correction according to clinical significance. These results were presented in HRs with 95% confidence intervals (CIs). The nonlinear relationship between TC and survival was tested by Cox regression model with Restricted Cubic Spline (RCS) regression, with 5-knot fitting correlation was applied. Based on the RCS Cox model, we generated a predicted risk score for each patient and evaluated the accuracy for survival outcomes at different time points using time-dependent receiver operating characteristic (ROC) curves. Subgroup analysis was conducted to analyze the association between TC and survival as a continuous variable in the groups of age (≤60 and >60 years), sex, BMI (<18.5, 18.5 ~ 23.9, >23.9 kg/m^2^), MSA subtypes (parkinsonian and cerebellar, abbreviated as MSA-P and MSA-C), diabetes, and hypertension. Statistical analyses were performed with SPSS (version 25.0) and R software (version 4.2.2), along with MSTATA software.^1^ All *p*-values were 2-tailed, and the statistical significance was set at <0.05.

## Results

### Clinical and hematological characteristics between different groups of serum TC at baseline

We subsequently divided patients with MSA into three groups according to serum TC tertiles (Q1, <3.91 mmol/L; Q2, 3.91 ~ 4.75 mmol/L; Q3, >4.75 mmol/L). As shown in Table 1, 183 (50.27%) patients were female, and 191 (54.27%) patients were MSA-C subtypes. The mean age and age of onset at baseline were 60.05 ± 8.52 and 58.39 ± 8.49 years, respectively. The mean disease duration at baseline was 1.66 ± 0.77 years. There were no significant differences in subtypes, age, age of onset, BMI, disease duration, score of UMSARS-II, score of UMSARS-IV, total UMSARS score, OH, smoking and drinking status between three groups according to the serum TC tertiles. Of note, the group 1 (Q1, <3.91 mmol/L) had lower proportion of female (43, 34.68% vs. 70, 59.83% vs. 70, 56.91%, *p* < 0.001), higher UMSARS-I score (14.83 ± 6.86 vs. 13.40 ± 6.39 vs. 12.32 ± 6.18, *p* = 0.010) and higher proportion of comorbidity of diabetes (29, 23.39% vs. 14, 11.97% vs. 13, 10.57%, *p* = 0.019). In the hematological characteristics (Table 2), we found that the group 1 (Q1, <3.91 mmol/L) had lower levels of albumin and other lipid markers (TG, HDL-C, and LDL-C). We failed to find the association between serum TC levels and other hematological index in this cohort.

**Table 1:** Baseline demographics in patients with MSA stratified by serum TC tertiles.

Characteristic	TC tertiles (mmol/L)			*P*-value
	Q1 (<3.91)	Q2 (3.91 ~ 4.75)	Q3 (>4.75)	
Number of patients	124	117	123	
Disease type (MSA-C, %)	67 (54.03%)	58 (49.57%)	66 (53.66%)	0.746
Age, years	61.44 ± 8.93	59.00 ± 8.74	59.65 ± 7.73	0.068
Age of onset, years	59.67 ± 8.96	57.42 ± 8.62	58.02 ± 7.74	0.101
Sex (female, %)	43 (34.68%)	70 (59.83%)	70 (56.91%)	<0.001*
BMI	23.70 ± 3.23	23.09 ± 3.28	23.39 ± 3.20	0.346
Disease duration, years	1.77 ± 0.79	1.58 ± 0.72	1.64 ± 0.78	0.138
UMSARS-I	14.83 ± 6.86	13.40 ± 6.39	12.32 ± 6.18	0.010*
UMSARS-II	16.60 ± 7.47	16.67 ± 6.79	15.46 ± 6.45	0.310
UMSARS-IV	1.97 ± 0.93	1.92 ± 0.91	1.82 ± 0.86	0.424
Total UMSARS	31.43 ± 13.60	30.07 ± 12.42	27.77 ± 11.87	0.074
OH (%)	41 (37.27%)	32 (29.91%)	27 (22.88%)	0.060
Smoking status				0.092
Never	71 (57.26%)	84 (71.79%)	85 (69.11%)	
Former	13 (10.48%)	12 (10.26%)	12 (9.76%)	
Now	40 (32.26%)	21 (17.95%)	26 (21.14%)	
Drinking status				0.382
Never	80 (64.52%)	78 (66.67%)	88 (71.54%)	
Former	9 (7.26%)	14 (11.97%)	11 (8.94%)	
Now	35 (28.23%)	25 (21.37%)	24 (19.51%)	
Comorbidity				
Diabetes (%)	29 (23.39%)	14 (11.97%)	13 (10.57%)	0.019*
Hypertension (%)	29 (23.39%)	20 (17.09%)	29 (23.58%)	0.382

*Significant difference. TC, total cholesterol; BMI: body mass index; UMSARS, Unified Multiple System Atrophy Rating Scale; OH, orthostatic hypotension.

**Table 2:** Baseline hematological characteristics in patients with MSA stratified by TC tertiles.

Characteristic	TC tertiles (mmol/L)			*P*-value
	Q1 (<3.91)	Q2 (3.91 ~ 4.75)	Q3 (>4.75)	
Number of patients	124	117	123	
ALT, U/L	23.91 ± 19.09	20.90 ± 14.07	26.30 ± 37.17	0.265
Albumin, g/L	42.63 ± 4.09	43.02 ± 3.34	44.54 ± 3.42	<0.001*
AST, U/L	22.30 ± 10.05	22.21 ± 10.18	24.74 ± 23.13	0.365
TBil, μmol/L	12.71 ± 5.98	11.75 ± 4.39	11.71 ± 5.30	0.243
BUN, μmol/L	6.32 ± 5.69	5.25 ± 1.47	5.88 ± 5.84	0.224
Scr, mg/dL	69.35 ± 27.71	66.39 ± 15.17	66.87 ± 13.34	0.461
eGFR, mL/min/1.73m^2^	89.22 ± 18.62	92.36 ± 13.64	91.78 ± 14.27	0.320
UA, μmol/L	307.47 ± 97.66	295.89 ± 69.84	297.94 ± 81.64	0.519
FBG, mmol/L	5.36 ± 1.81	5.24 ± 1.06	5.19 ± 0.84	0.588
TG, mmol/L	1.19 ± 0.88	1.37 ± 0.67	1.68 ± 0.81	<0.001*
HDL-C, mmol/L	1.30 ± 0.47	1.33 ± 0.30	1.42 ± 0.42	0.042*
LDL-C, mmol/L	1.75 ± 0.37	2.53 ± 0.32	3.39 ± 0.55	<0.001*

*Significant difference. TC, total cholesterol; ALT, alanine aminotransferase; AST, aspartate aminotransferase; TBil, Serum total bilirubin; BUN, blood urea nitrogen; Scr, creatinine; eGFR, estimated glomerular filtration rate; UA, uric acid; FBG, fasting blood glucose; TG, triglyceride; HDL-C, low-density lipoprotein cholesterol; LDL-C, high-density lipoprotein cholesterol.

### Survival analysis and predictive ability of serum TC in patients with MSA

**Figure 1:**
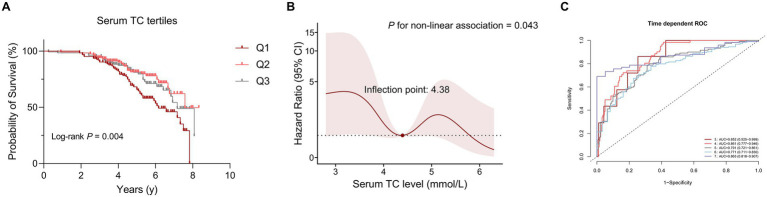
The correlation between serum total cholesterol (TC) levels and survival in patients with multiple system atrophy (MSA). **(A)** Comparison of survival between the three groups according to serum TC tertiles. **(B)** Association between serum TC (mmol/L) and survival using a multivariable-adjusted Restricted Cubic Spline (RCS) model. **(C)** Time-dependent receiver operating characteristic (ROC) curves in predicting MSA mortality using RCS models.

Kaplan–Meier survival analysis was used to analyze the relationship between serum lipid levels and survival. As mentioned above, we divided patients with MSA into three groups according to the distribution of serum TC. The survival duration was significantly different among the three groups of TC (Log-rank *p* = 0.004) (Figure 1A). According to the tertiles of TC, the survival duration of patients in group 1 (the lowest group of serum TC level) was shorter than those in group 2 (the intermediate group of serum TC level) (mean survival time: 6.2 vs. 7.6 years, log-rank *p* = 0.012) and group 3 (the highest group of serum TC level) (mean survival time: 6.2 vs. 7.2 years, log-rank *p* = 0.025). The unadjusted Cox analysis demonstrated significantly decreased risk of mortality across increasing TC [HRs and 95% CIs: 0.70, (0.57–0.86), *p* < 0.001] (Supplementary Figure 1). Although multivariate Cox analysis using TC as a continuous variable revealed no significant association with survival (Supplementary Figure 1), analysis using TC as a categorical variable demonstrated that the level of TC in group 2 decreased the risk of mortality of patients with MSA compared to the level of TC in group 1, after adjusting for age, sex, subtype, BMI, disease duration, total UMSARS score, OH, diabetes, hypertension, albumin, TG, HDL-C and LDL-C (HRs and 95% CIs of group 2: 0.47, [0.23–0.96]; group 1 for reference; *p* = 0.038). Additionally, after adjusting for age, sex, subtype, BMI, disease duration, total UMSARS score, OH, diabetes, hypertension, albumin, TG, HDL-C and LDL-C, the RCS analysis showed a non-linear relationship between serum TC level and survival with the lowest risk of death at the value of 4.38 mmol/L (Figure 1B). According to the predicted risk score, the time-dependent ROC analysis revealed discriminatory performance in various time points, with area under the curve (AUC) values of 0.852 (95% CI: 0.525–0.999) at 3 years, 0.861 (95% CI: 0.777–0.946) at 4 years, 0.791 (95% CI: 0.721–0.861) at 5 years, 0.771 (95% CI: 0.711–0.830) at 6 years, 0.863 (95% CI: 0.818–0.907) at 7 years (Figure 1C). This indicates that the model incorporating the nonlinear effect of serum TC effectively predicted mortality in MSA (Table 3).

**Table 3:** Univariate and multivariate Cox proportional-hazards regression analyses for survival according to serum TC level.

Characteristic	Univariate		Multivariate	
	HR (95% CI)	*P*-value	HR (95% CI)	*P*-value
Serum TC (categorized) ^a^	Ref	Ref	Ref	Ref
	0.49 (0.30, 0.79)	0.003*	0.47 (0.23, 0.96)	0.038*
	0.59 (0.38, 0.91)	0.018*	1.02 (0.36, 2.89)	0.978

^*^Significant difference. ^a^multivariate model adjusted by age, sex, subtype, BMI, disease duration, total UMSARS score, OH, diabetes, hypertension, albumin, TG, HDL-C and LDL-C; TC, total cholesterol; TG, triglyceride; LDL-C, low-density lipoprotein cholesterol; HDL-C, high-density lipoprotein cholesterol.

### Subgroup analysis

**Figure 2:**
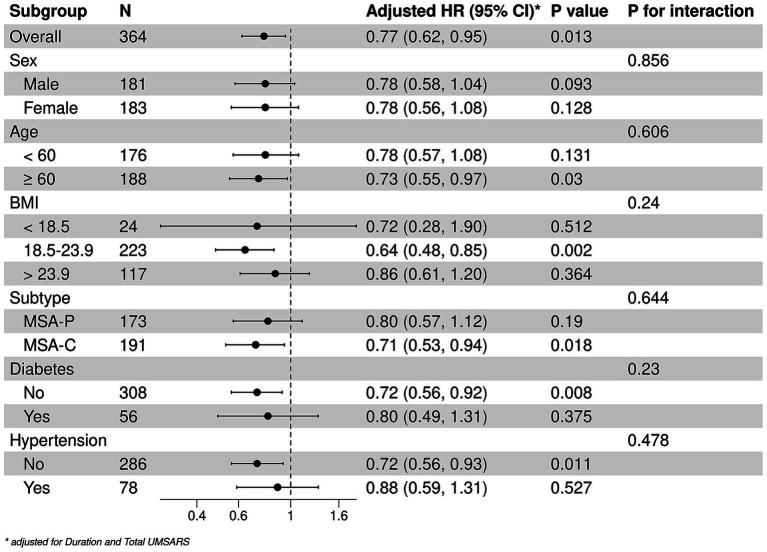
Stratified analysis of the associations between serum TC and survival. *Adjusted by disease duration and total UMSARS scores.

The stratified analyses of the predictive level of serum TC (continuous) on the mortality in patients with MSA according to age, sex, BMI, MSA subtype, diabetes, and hypertension. A negative correlation between TC and mortality were detected among patients older than 60 years [HRs and 95% CIs: 0.73, (0.55–0.97), *p* = 0.003], those with normal BMI [HRs and 95% CIs: 0.64, (0.48–0.85), *p* = 0.002], MSA-C [HRs and 95% CIs: 0.71, (0.53–0.94), *p* = 0.018], without hypertension [HRs and 95% CIs: 0.71, (0.56–0.93), *p* = 0.011] or diabetes [HRs and 95% CIs: 0.72, (0.56–0.92), *p* = 0.003]. In the subgroup analysis, the effect of serum TC levels on mortality of patients with MSA was consistent in these subgroups (Figure 2).

## Discussion

This study explored the relationship between serum TC and survival of patients with MSA in the early stages. Our findings indicated that the serum TC was a significant factor to impact the survival of patients with MSA. Patients with lower levels of serum TC had greater risk of mortality compared to those with higher level of serum TC. Moreover, the RCS model suggested that the serum TC value of 4.38 mmol/L was the threshold for the lower risk of mortality of patients with MSA. A negative trend was observed in the mortality of patients with MSA as the level of serum TC lower than 4.38 mmol/L. The risk score from RCS Cox model also demonstrated an effective prediction in mortality over years.

Our cohort study firstly explored the prognostic implications of serum TC levels in patients with MSA in the early stage. Given that elevated serum TC is associated with vascular risk factors, we propose that maintaining serum TC levels lower than 5.0 mmol/L may represent a more beneficial lifestyle for patients with MSA. Our previous cohort and other cross-sectional studies demonstrated that serum TC levels were lower in patients with MSA than in healthy controls, suggesting that reduced serum TC levels increase the risk of MSA (13, 14). In addition, subgroup analyses revealed that in patients with MSA over the age of 60 years, the serum TC levels were correlated with survival. Previous studies have indicated that lipid metabolism disorders with the changes of TC levels, occur with aging in healthy elderly populations (22). It has been observed that serum TC levels tend to decline in individuals over 60 years old (23, 24). Consequently, we speculated that the low serum TC levels may exacerbate the pathophysiological mechanisms associated with aging, thereby worsening the progression of MSA. We did not identify the relationship between the serum TC levels and survival in patients with MSA who were classified as overweight or underweight. Notably, patients with a high BMI exhibited a greater prevalence of dyslipidemia, while low body weight group with worse nutritional status. These factors may influence the relationship between serum TC and survival in these two groups of MSA. Additionally, we observed a significant negative correlation between the level of serum TC levels and mortality in patients with MSA-C. Serum TC levels were also negatively correlated with the risk of OH in patients with MSA (15). These findings suggested that cerebellar pathophysiology and autonomic nervous system dysfunction may be particularly vulnerable to the disturbances in serum lipid levels, especially the serum TC (25).

Cholesterol serves as a critical component of cytoplasmic membranes, including the myelin sheath within the central nervous system (26). Given that *α*-synuclein aggregates with oligodendrocyte involvement as the main pathological feature of MSA (27), disruptions of cholesterol synthesis and metabolism may influence the abnormal development of oligodendrocytes and the formation of myelin (28–30). Current research suggests that deficiencies in the myelin sheath are associated with α-synuclein aggregation (31, 32). In addition, phosphatidylcholine, which predominantly composed of cholesterol, interacts with α-synuclein monomers, facilitating the formation and stabilization of toxic α-synuclein oligomers (33). Notably, knockout specific upstream genes responsible for cholesterol production in oligodendrocytes *in vivo* induced ataxia and dyskinesia in murine models (34), underscoring the essential role of cholesterol in maintaining the physiological functions in oligodendrocytes.

Some limitations remained in the study. First, the investigation was confined to measuring cholesterol levels in peripheral blood, which may not accurately reflect cholesterol levels within the central nervous system. Consequently, further research is warranted to determine whether serum cholesterol levels can serve as a reliable indicator of cholesterol metabolism in neurons, oligodendrocytes, and other glial cells. Second, the study was conducted as a single-center prospective investigation, primarily representing patients from western China. Additionally, while the patients included in the study met the clinical criteria for MSA, none had undergone autopsy confirmation. Considering these limitations, multicenter collaborations with extended follow-up periods and pathological diagnoses are needed to be carried out to validate our findings.

## Conclusion

Our prospective cohort study indicated that low serum TC levels are associated with an increased risk of mortality in patients with MSA, exhibiting a non-linear form. The association between serum TC levels and survival was particularly pronounced among the elderly patients and those with the MSA-C subtype. Abnormal cholesterol metabolism may represent a significant pathophysiological factor influencing the survival of MSA.

## Data Availability

The raw data supporting the conclusions of this article will be made available by the corresponding author, on reasonable request.

## Footnotes

^1^ www.mstata.com

## Supplementary material

Supplementary Figure 1 Univariate and multivariate Cox regression model proportional-hazards regression analyses for MSA survival according to baseline characteristics.
