# Supplementary figures and images for: Low level of serum total cholesterol predicts mortality in early-stage multiple system atrophy: a prospective-cohort study
Source: Front Nutr. 2025 Oct 28;12:1663881. doi: 10.3389/fnut.2025.1663881 (PMC12602236; doi:10.3389/fnut.2025.1663881)

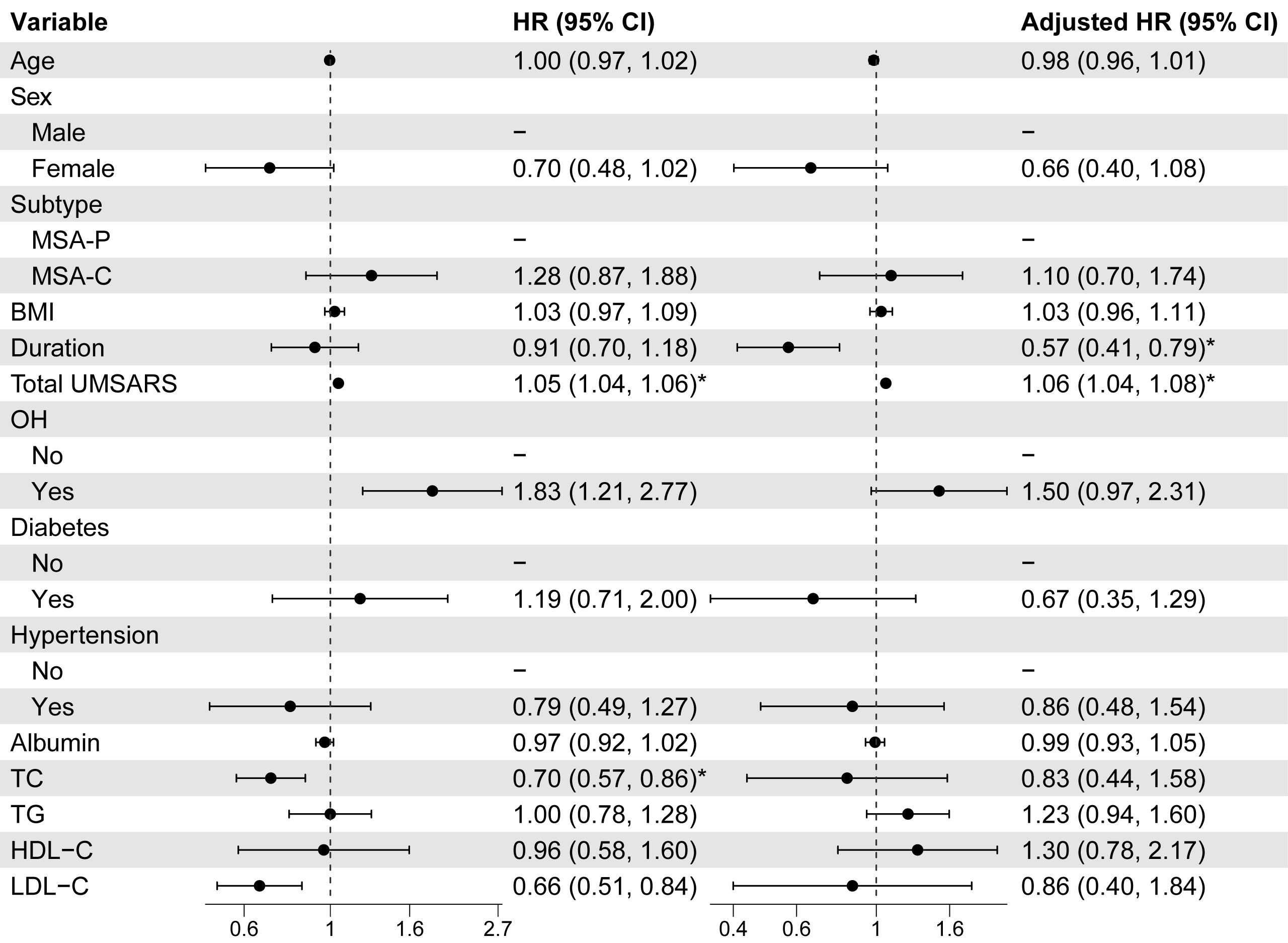

Supplement: Supplementary Figure 1 — Univariate and multivariate Cox regression model proportional-hazards regression analyses for MSA survival according to baseline characteristics. [file Image_1.tif]
